# Supplementary material for: Resolving the full spectrum of human genome variation using Linked-Reads
Source: Genome Res. 2019 Apr;29(4):635–45. doi: 10.1101/gr.234443.118 (PMC6442396; doi:10.1101/gr.234443.118)
Supplement: Supplemental Material [file supp_gr.234443.118_Supplemental_longranger-2.2.zip › longranger-2.2.tar.bz2!/lr2.2-public/lib/stan/cmdstan/stan/doc/api/html/check__column__index_8hpp_source.html]

Stan: src/stan/math/error\_handling/matrix/check\_column\_index.hpp Source File


|  |  |
| --- | --- |
|  | Stan  2.5.0  probability, sampling & optimization |


- Main Page
- Related Pages
- Namespaces
- Classes
- Files

- File List
- File Members

All Classes Namespaces Files Functions Variables Typedefs Enumerations Enumerator Friends Macros Pages

- src
- stan
- math
- error\_handling
- matrix

check\_column\_index.hpp

Go to the documentation of this file.

1 #ifndef STAN\_\_MATH\_\_ERROR\_HANDLING\_\_MATRIX\_\_CHECK\_COLUMN\_INDEX\_HPP

2 #define STAN\_\_MATH\_\_ERROR\_HANDLING\_\_MATRIX\_\_CHECK\_COLUMN\_INDEX\_HPP

3

4 #include <sstream>

5 #include <stan/math/error\_handling/dom\_err.hpp>

6 #include <stan/math/matrix/Eigen.hpp>

7

8 namespace stan {

9  namespace math {

10

24  template <typename T\_y, typename T\_result, int R, int C>

25  inline bool check\_column\_index(const char\* function,

26  size\_t i,

27  const Eigen::Matrix<T\_y,R,C>& y,

28  const char\* name,

29  T\_result\* result) {

30  if ((i > 0) && (i <= static\_cast<size\_t>(y.cols())))

31  return true;

32

33  std::ostringstream msg;

34  msg << name << " (%1%) must be greater than 0 and less than "

35  << y.cols();

36  std::string tmp(msg.str());

37  return dom\_err(function,i,name,

38  tmp.c\_str(),"",

39  result);

40  }

41

42  }

43 }

44 #endif

dom\_err.hpp

stan::math::dom\_err

bool dom\_err(const char \*function, const T &y, const char \*name, const char \*error\_msg, const T\_msg error\_msg2, T\_result \*result)

**Definition:** dom\_err.hpp:36

stan::math::check\_column\_index

bool check\_column\_index(const char \*function, size\_t i, const Eigen::Matrix< T\_y, R, C > &y, const char \*name, T\_result \*result)

Return true if the specified index is a valid column of the matrix.

**Definition:** check\_column\_index.hpp:25

Eigen.hpp


---

[ Stan Home Page ]

*© 2011–2014,
Stan Development Team.*
